# Integrating pathology and radiology disciplines: an emerging opportunity?

**DOI:** 10.1186/1741-7015-10-100

**Published:** 2012-09-05

**Authors:** James Sorace, Denise R Aberle, Dena Elimam, Silvana Lawvere, Ossama Tawfik, W Dean Wallace

**Affiliations:** 1 Office of Science and Data Policy, Office of the Assistant Secretary for Planning and Evaluation, US Department of Health and Human Services, 200 Independence Ave., SW Washington, D.C. 20201, USA; 2 Department of Radiological Sciences, David Geffen School of Medicine at UCLA, UCLA Medical Center, Box 951721, Los Angeles, CA 90095-1721, USA; 3 SciMetrika, LLC, 100 Capitola Drive, Suite 106, Durham, NC 27713-4451, USA; 4 SciMetrika, LLC, 100 Capitola Drive, Suite 106, Durham, NC 27713-4451, USA; 5 Department of Pathology and Laboratory Medicine, Kansas University Medical Center, 3901 Rainbow Blvd., Mail Stop 3045, 2017 Wahl Hall West, Kansas City, KS, 66160-7410, USA; 6 Department of Pathology, David Geffen School of Medicine at UCLA, 10833 Le Conte Avenue, Center for Health Sciences, Room 13-222, Los Angeles, CA 90095-1672, USA

**Keywords:** Pathology, Radiology, Quality Improvement, Health Information Technology, Standards, Interoperability

## Abstract

Pathology and radiology form the core of cancer diagnosis, yet the workflows of both specialties remain *ad hoc *and occur in separate "silos," with no direct linkage between their case accessioning and/or reporting systems, even when both departments belong to the same host institution. Because both radiologists' and pathologists' data are essential to making correct diagnoses and appropriate patient management and treatment decisions, this isolation of radiology and pathology workflows can be detrimental to the quality and outcomes of patient care. These detrimental effects underscore the need for pathology and radiology workflow integration and for systems that facilitate the synthesis of all data produced by both specialties. With the enormous technological advances currently occurring in both fields, the opportunity has emerged to develop an integrated diagnostic reporting system that supports both specialties and, therefore, improves the overall quality of patient care.

## Background

Pathology and radiology form the core of cancer diagnosis. Pathology characterizes the specific histologic and molecular features of tissues, while radiology localizes suspicious lesions and informs clinical-stage and potential comorbidity determinations. Under the current paradigm of diagnostic medicine, pathologists and radiologists function as members of distinct disciplines, with no direct linkage between their workflows or reporting systems. Even when both departments belong to the same institution, their respective reports on the same patient are only loosely associated with one another by identifiers such as patient's name and medical record number. Despite this complete bifurcation of reporting, the synthesis of both specialties' data must establish diagnosis, determine prognosis, drive patient management and serve as the primary means for assessing response to treatment. Unfortunately, current practice of reviewing pathologists' and radiologists' reports is limited to hospital tumor boards that do not typically review patients with negative pathological findings, and all too often the responsibility for correlation falls on the clinician ordering the study. Consequently, a radiology and pathology diagnostic reporting system that integrates text, sentinel images and molecular diagnostic data to an integrated, coherent interpretation would better inform management decisions.

## Discussion

### Documented disease-specific needs for radiology-pathology integration in diagnostic medicine

Several studies have identified needs for the integration of mammography and pathology reporting in the setting of specific breast cancer diagnosis, where integration and correlation between the two specialties was shown to detect misdiagnosis and to prompt repeat biopsy in instances of unexplained discrepancy between imaging and pathologic findings [1-3]. Studies have also shown that cooperation between pathologists and radiologists is critical in some situations, for example, when sentinel lymph nodes must be localized and when targeted lesions and their resection margins must be localized and mapped to determine the adequacy of the resection. This kind of cooperation can both prevent the need for re-excision and improve the reporting of survival-related prognostic parameters [4,5].

Recently, the benefits of pathology-radiology integration have become apparent in other clinical settings. For example, the new classification of lung adenocarcinoma by the International Association for the Study of Lung Cancer, the American Thoracic Society, and the European Respiratory Society acknowledges that an integrated, multidisciplinary approach to lung adenocarcinoma is required for optimal patient care [6]. Factors that drove this conclusion include specific molecular signatures in adenocarcinoma that have led to specific, targeted therapies [7]. In addition, lesion characterizations by high-resolution helical computed tomography have introduced a new classification of peripheral lung lesions, such as ground-glass and part-solid nodules, that correlate with histologic features and inform prognosis and therapy.

Similar diagnostic benefits from pathology-radiology integration have been observed with regard to interstitial lung disease. Historically, the diagnosis of interstitial lung disease has been the domain of the pulmonologist, who is required to independently correlate the histopathologic report and radiographic findings to be able to identify prognostically distinct subsets [8,9]. Recently, however, the American Thoracic Society and the European Respiratory Society have issued international consensus statements that call for an integrated, dynamic interaction among clinician, imager and pathologist to better inform the specific biological behavior and prognosis of subsets of interstitial lung disease [10-12]. Three reasons motivated this call for change:

▪ different patterns of interstitial lung disease can coexist in different sites, so tissue sampling error may be misleading in cases of such heterogeneity;

▪ an integrated report that includes both the histologic pattern of disease and the clinical and imaging features can provide more accurate diagnostic and prognostic information; and

▪ some cases of interstitial lung disease are histopathologically indeterminate even for the most experienced and specialized lung pathologists [13].

Moreover, studies of interstitial lung disease have shown that such multidisciplinary interactions increase interobserver agreement [14,15].

As with breast cancer and lung disease, the domains of bone and soft-tissue disease diagnoses, including vascular tumors and malformations, require pathology-radiology integration. The anatomic location and radiographic features of a bone or soft-tissue tumor, together with the histologic morphology, determine the type and nature of a specific tumor. Without integration of the diagnostic findings, both the individual radiology and individual pathology reports remain incomplete in the clinical setting [16,17]. Finally, pathology-radiology integration may improve the classification and diagnosis of vascular tumors and malformations [18].

### The general benefits of pathology-radiology integration

Beyond the documented need for improved management of specific diseases, many potential benefits would derive from pathology-radiology integration and, therefore, argue for its universal adoption. These include:

▪ **Report standardization**. Multidisciplinary collaborations foster the use of controlled terminologies and standardized reporting structures, making evaluation across multiple patient cohorts possible.

▪ **Clinical efficiency**. Diagnostic radiology-pathology integration would yield reporting that is accessible via a single portal, which could free more time for interpretation and decision making.

▪ **Registry development**. The aggregation of diagnostic imaging and pathology data into a single resource would facilitate the development of rich databases for disease registries, which would enable or improve:

- quality assurance,

- identification of potential research cohorts,

- research data mining, and

- efficient access by tumor registries.

▪ **Tumor board presentation**. The integrated report may lessen preparation time for multidisciplinary tumor board conferences and would be a valuable educational resource for medical students and residents.

▪ **Research and innovations**. Recent advances in both pathology and radiology, are encouraging more cooperation between the two disciplines in situations in which tumors can be identified with certainty at an earlier stage than in the past and then characterized at the molecular level. Rapid access to comprehensive pathology and radiology findings will be necessary to enable novel translational research activities, including, for example, the use of a multidisciplinary Sequencing Tumor Board as a means to assess the molecular findings of specific patients and make recommendations for their treatment [19].

▪ **Improved patient care**. Diagnostic radiology-pathology integration would improve patient care. In addition to the benefits noted above, greater coordination would encourage the use of less invasive techniques, such as needle biopsies, while monitoring each event to assure that adequate material is available for additional studies, including molecular diagnostics.

Consequently, between the imaging and pathology worlds, a movement is growing to develop systematic processes that can exploit both the integration of diagnostic information and the flow of small tissue samples obtained from image-guided biopsy from radiology to pathology. This multidisciplinary effort has led to a number of recommendations for pathologic and molecular diagnosis, as well as radiologic practice.

### The limitations of current workflows and the challenge of the installed base

Many issues must be resolved before an integrated pathology-radiology workflow can be adopted. The two disciplines rely on numerous legacy information systems. Within pathology departments, for example, anatomical pathology and clinical pathology frequently use different information systems to track specimens and produce reports. Anatomic pathology may also use a third system to view and store pathology images. Similarly, radiologists use a picture archiving and communication system to disseminate and archive images and a radiology information system to archive text reports. In response to the unique needs of mammography screening and to ensure compliance with the Mammography Quality Standards Act, radiologists often also use a dedicated mammography information system.

Moreover, for most intra-institutional communications between radiology and pathology to occur, the radiology and pathology systems must communicate not only with one another, but also with other systems, including the hospital information system, the laboratory information system and electronic medical records. This extent of integration frequently requires expensive custom interfaces. Aside from the cost of development, where an exchange has been developed, implementation is typically incomplete. For example, current implementations fail to support the advanced Health Level 7 features required to support anatomical pathology test-ordering and reporting functions. In addition, the lack of integration between commercially available information systems prevents a unified view of a patient's records, resulting in the need to query multiple radiology and pathology systems. This situation becomes even more complicated in settings where the pathologist and radiologist do not share a common information technology infrastructure (for example, interface with a common hospital Electronic Health Record (EHR)). Many of these issues can be resolved through the adoption of appropriate standards by system vendors and end users.

### Advances in standards and technology that enable integration

Although the installed base has significant shortcomings, some emerging standards and evolving pathology and imaging technologies nonetheless present opportunities to integrate radiology and pathology information.

#### Emerging standards in pathology and radiology

The following emerging standards have significant potential to resolve some of the current workflow challenges:

▪ The College of American Pathologists' Electronic Cancer Checklists (CAP eCC) are used to report malignant findings in a structured format [20]. The goal of the CAP eCC is to codify pertinent diagnostic findings while meeting the requirements of the American Joint Committee on Cancer staging.

▪ Until recently, the field of digital pathology had been hindered by the lack of standards for storing and transferring images. However, this issue was addressed when the Digital Imaging and Communications in Medicine supplement for digital pathology (Supplement 145)^i ^was released in July 2010 [21].

▪ Many radiology groups are voluntarily implementing the Radiological Society of North America's structured reporting templates [22]. Although the structured reports must be customized to the needs of the particular referring physician (for example, emergency medicine reporting differs substantially from medical oncology reporting), structured templates were found to improve patient care by increasing clarity and thoroughness in the communication of imaging findings [23].

▪ RadLex is an evolving controlled terminology for radiology reporting, teaching and research. Originally designed to standardize terminologies only in chest imaging, it now covers several facets of radiology, with the following goals:

- to reduce interpretation variability,

- to map synonyms that refer to the same concepts,

- to provide a terminology comprehensible for both humans and computers,

- to facilitate data mining and querying of reports,

- to automate image annotation and computer-aided diagnosis, and

- to enable teaching files and decision support [24,25].

#### Technological advances in pathology

Two sets of emerging technologies in the field of pathology could, especially when combined, lead to much more efficient and thorough diagnoses and prognoses. First, molecular diagnostic techniques have led to the incorporation of prognostic and predictive molecular biomarkers for the benefit of patient management. Second, digital imaging with supervised, quantitative image analysis will improve the informational content of integrated reports and link users directly to digital images of the pathology slides. Quantitative image analysis will also permit more reproducible evaluation of the morphologic findings, specifically, expression of markers by immunohistochemistry. The benefits of combining these sources of information into the pathology section of an integrated pathology-radiology report include enhancement of the pathology report itself and improved facilitation of conference preparation and medical education; it would also reduce the chance that these findings might be overlooked, a risk that is increased if they are reported as separate ancillary studies.

#### Technological advances in radiological imaging

As with pathology, radiology has two emerging technologies that invite integration. Now possible is the incorporation of the functional and physiologic information from magnetic resonance and positron emission tomography (PET) into radiology reports. PET scans, for example, decrease the rate of biopsies in masses that are not deemed active by imaging characteristics. Secondly, semi-automated quantitative image analysis is also now available and amenable to integration into the radiology report. Evidence is accumulating that computerized image analysis improves radiologists' diagnostic performance in terms of both the detection and the characterization of diseases. Moreover, this advanced image analysis has been shown to improve diagnostic performance across a spectrum of diseases by reducing reader variability and providing reproducible quantification of features, such as tumor volume, consistency and perfusion, yielding reliability not possible with visual inspection alone [26-28]. These data are more valuable as complements to histology when contextualized by a fully integrated report. In addition, when integrated, both technologies have potential to improve the assessment of treatment response and tracking of disease evolution.

## Summary

### The path forward: critical components of radiology-pathology integration

**Figure 1:**
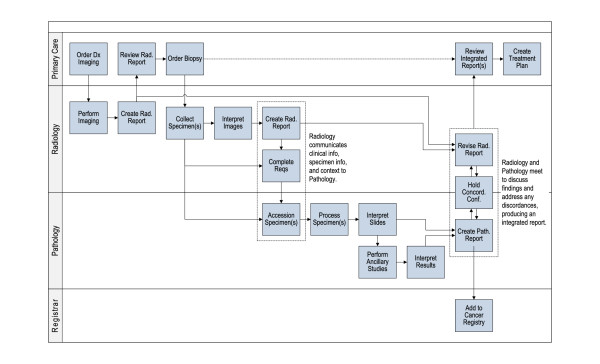
**Proposed idealized workflow model of radiology-pathology integration**. Workflows are not designed around any specific disease; they remain flexible to meet new clinical demands. This figure is from the US Health and Human Services Report titled "*The importance of radiology and pathology communication in the diagnosis and staging of cancer: mammography as a case study" *(manuscript reference 3) and is thus not copyrighted.

The formal process of correlating radiology and pathology, including the timely resolution of discordance, must be supported. To this end, pathology-radiology integration workflows must ensure the flow of communications and specimens and link structured diagnostic results from pathologists with those of radiologists. These processes can be either asynchronous (that is, intermittent e-mail-like exchanges) or synchronous (that is, conferences). The workflow in Figure 1 proposes an idealized model that maximizes pathology-radiology integration and identifies key roles, including the referring clinician, the radiologist, the pathologist and other users of radiology-pathology information, such as cancer registries.

To enable the extent of the integrated diagnostic consultation modeled in Figure 1, the following challenges must be addressed:

• Structured reporting that includes the use of controlled vocabularies is required to enable consistent information content within both domains and better automate concept extraction and database population.

• The specialties will require national practice guidelines to aid identification of possible discordant pathology-radiology results. In addition to documenting and correlating positive pathology-radiology findings, these guidelines should address the reporting of benign or negative pathology results that become especially problematic if benign pathology results are discordant with radiology findings and which may, therefore, cause an incorrect patient diagnosis.

• Vendors will have to act on these emerging opportunities by developing comprehensive management systems that consider clinical workflow and support the integration of textual, image and quantitative data generated by both disciplines. Doing so will provide a coherent and meaningful consultation resource and data repository.

• The specialties must document the benefits of integrated consultation on the bases of clinician feedback and measurable changes in the efficiency and accuracy of diagnosis, treatment timing and, ultimately, patient outcomes.

• The specialties must achieve interoperability in situations where radiology and pathology are housed in different institutions.

Despite technical challenges that limit integration within current workflow models, the opportunity for pathology-radiology integration to improve patient care is great, and more importantly, the tools to achieve this end exist. Institutions, such as Kansas University Medical Center and the University of California, Los Angeles, have already begun implementing different models of integration [3,29,30]. Other opportunities for achieving integration involve developing models of pathology and radiology collaboration that foster joint training and are supported by innovative payment models. Member organizations can play an important role in fostering educational exchanges between the disciplines. Recently, for example, the American College of Radiology launched the American Institute for Radiologic Pathology. The institute offers a Radiologic-Pathologic Correlation course to radiology residents to provide them with an opportunity to learn the pathologic counterpart to their findings and emphasizes the correlation of pathology and the medical images [31]. Implementing similar programs for both specialties during medical training and as continuing-education opportunities would likely foster radiology-pathology integration on a wider scale.

## Abbreviations

CAP eCC: College of American Pathologists' Electronic Cancer Checklists; DICOM: Digital Imaging and Communications in Medicine; EHR: Electronic Health Record; PET: Positron Emission Tomography; RadLex: Radiological Society of North America's Radiology Lexicon

## Competing interests

JS, DRA, DE, SL, OT and WDW declare they have no competing interests.

## Authors' contributions

JS, DRA, DE, SL, OT and WDW contributed equally to the development of this commentary, including in its conception, intellectual development and drafting of the final version. All authors have read and approved the final manuscript.

## Pre-publication history

The pre-publication history for this paper can be accessed here:

http://www.biomedcentral.com/1741-7015/10/100/prepub

## Endnotes

This supplement will become part of the DICOM PS 3 Standard, which is under continuous maintenance by National Electrical Manufacturers Associations (NEMA). The current official version of the standard is available at ftp://medical.nema.org/medical/dicom/2009/.
